# Psychometric properties of Haj-Yahia’s questionnaire of violence against women in a sample of married women in Tehran, Iran

**DOI:** 10.1186/s12889-022-12831-8

**Published:** 2022-03-07

**Authors:** Sahar Sotoodeh Ghorbani, Mohtasham Ghaffari, Seyed Saeed Hashemi Nazari

**Affiliations:** 1 Department of Epidemiology School of Public health and Safety, Shahid Beheshti University of Medical Sciences, Tehran, Iran; 2 Environmental and Occupational Hazards Control Research Center, Department of Public Health, School of Public Health and Safety, Shahid Beheshti University of Medical Sciences, Tehran, Iran; 3 Prevention of Cardiovascular Disease Research Center, Department of Epidemiology, School of Public Health and Safety, Shahid Beheshti University of Medical Sciences, Tehran, Iran

**Keywords:** Psychometric, Wife abuse, Questionnaire, Women

## Abstract

**Background:**

Abuse against women causes great suffering for the victims and is an important health problem among women. To date, a few screening instruments for wife abuse exist for married women in Iran, but they only assess some of the wife abuse components. The aim of this study was to investigate the psychometric properties and factor structure of the Haj-Yahia’s Questionnaire in a sample of married women residing in Tehran.

**Materials and methods:**

This is a cross-sectional study with a population consisting of married women in Tehran, among which 471 individuals were selected using convenience sampling method. Psychometric properties of the questionnaire were evaluated using face validity, content validity, construct validity, internal consistency, and stability. Confirmatory factor analysis was performed using the weighted least square mean and variance adjusted. We performed confirmatory factor analysis using Mplus version 8 software and for other calculations, we used STATA V14.

**Results:**

The quantitative results of face validity and content validity indicated that all items of the questionnaire were in acceptable range, and were retained in the study. In CFA results, the model fit indices were acceptable (TLI = 0.986, CFI = 0.987, RMSEA = 0.039 and SRMR = 0.057). Cronbach’s alpha coefficient for psychological abuse, physical abuse, sexual abuse, and economic abuse were estimated 0.90, 0.93, 0.79, and 0.78 respectively, and an alpha of 0.95 was found for the total questionnaire. The intra-cluster correlation index was 0.98.

**Conclusions:**

Findings showed that the Persian version of the questionnaire of violence against women made it possible to evaluate various dimensions of violence using 4 factors and showed good construct validity and internal reliability in the female population in Iran; therefore, it can be used in future studies.

**Supplementary Information:**

The online version contains supplementary material available at 10.1186/s12889-022-12831-8.

## Background

Wife abuse is one of the most important types of domestic violence, through which men exercise their social or physical power on women. This type of violence is divided into three general categories including physical abuse, emotional abuse, and sexual abuse [1]. Violence against women as one of the major public health concerns in today’s world has turned into a pervasive phenomenon in recent decades [2]. According to the reports by the United Nations, the prevalence of wife abuse is 25% in Belgium, 28% in the United States, 25% in Norway, 17% in New Zealand, 38% in Korea, 20% in Colombia, and 58–67% in New Guinea [3]. The prevalence of wife abuse in Iran is in the range of 30 to 80%. The most accurate rate of wife abuse in the country was provided by the National Survey, which estimated it as 66% [4].

‌‌ Studies conducted in this area showed the high rate of wife abuse. Ahmadi et al. [5] indicated that 35% of married women were subjected to various forms of domestic violence, of which 30% were subjected to physical violence, 29% to psychological violence, and 10% to sexual violence. Violence may have non-fatal physical consequences from cuts to fractures and damage to internal organs, unwanted pregnancy, sexually transmitted diseases, unintended abortion, pelvic inflammatory disease, chronic pelvic pain, headache, irritable bowel syndrome, smoking, addiction, alcoholism, nutritional problems, and sexual disorders; fatal consequences such as suicide and murder, and psychological consequences such as depression, fear, anxiety, and obsession [6].

Most researches have focused on preventing problems caused by wife abuse. Early psychotherapy interventions in the event of domestic violence reduce women’s psychological problems [7]. Psychotherapy interventions include a range of interventions that target cognition, motivation, and behavior. These include (1) formal cognitive behavioral therapy (CBT) and trauma-focused CBT, and CBT‐based techniques; (2) integrative therapies including motivational interviewing; and (3) behavior therapies e.g. relaxation techniques; (4) humanistic therapies e.g. supportive and non‐directive therapies; (5) and other psychologically‐orientated interventions e.g. art therapy, meditation, and narrative therapy [8]. Therefore, due to the importance of identifying, diagnosing, preventing, and performing therapeutic interventions, a tool for measuring and evaluating wife abuse is needed [9].

In foreign research to measure and evaluate wife abuse, different measures such as the Conflict Tactics Scale-Revised (CTS-R), Abuse Assessment Screen, Violent Behavior Inventory, Emotional Violence Scale, and Violence against Women Questionnaire (Haj Yahya, 1999) are used. The psychometric properties of these tools have not been studied except for Conflict Tactics Scale-Revised (CTS-R) in Iran [9–15].

In Iran, in addition to the Conflict Resolution Tactics Questionnaire, Spouse Abuse Questionnaire (Ghahari et al.,2006), and the translated version of Questionnaire by Moffitt et al. (Shams Esfandabad and Emamipour, 2003) are commonly used to evaluate wife abuse [16–18]. However, reviewing these tools clarify limitations and shortcomings such as large number of questions, not covering all types of wife abuse, and the incompatibility of some questions of the questionnaire with the cultural and social environment. Also, some of these tools, including the Conflict Resolution Tactics questionnaire, do not specifically and comprehensively examine wife abuse.

Haj-Yahia’s questionnaire of Violence Against Women (1999) is adapted from the other five questionnaires, including the Conflict Tactics Scales (Straus, 1980), the Psychological Maltreatment of Women Inventory (Tolman, 1990), the Measure of Wife Abuse (Rodenberg & Fantuzzo, 1995), the Index of Spouse Abuse (Hudson & McIntosh, 1981), and the Abusive Behavior Inventory (Shepard & Campbell, 1992) [15]. This questionnaire is a combination of existing questionnaires and measures all aspects of wife abuse. Due to the importance and necessity of this tool to be used in various researches, the present study was conducted to the psychometric evaluation of the Persian version of Haj-Yahia’s questionnaire of Violence against Women in a sample of married women in Tehran, Iran.

## Materials and methods

### Study design and participants

The present study is a cross-sectional, descriptive-analyzing one, which started in February 2020 and ended in June 2020. Considering the 41.7% prevalence of violence against women [19], and 5% error, the sample size of 374 people was estimated. Eventually, considering 20% non-response, 471 samples were selected. Samples were selected through convenience sampling. To do so, the questionnaire was designed electronically and then shared on Telegram channels and WhatsApp groups. Married women living in Tehran were asked to complete the questionnaire if they wished to participate in this study. Inclusion criteria were defined as to be married women living in Tehran with at least one year of the marriage record. Women, who were widowed, divorced, or living apart from their husbands, were excluded from the study. Out of 471 participants, 42.04% were employed and 57.96% were housewives. The age range of the subjects was between 18 and 58, with a mean and standard deviation of 32.69 ± (7.9) years. The subjects hold associate and bachelor’s degrees (50.96%), diploma and lower level’s certificate (27.60%), and Masters’ and higher degrees (21.44%), respectively. The couples have been married for an average of 9.48 years, with a minimum of 1 and a maximum of 39 years. 91.3% of these women have chosen to marry and 8.7% have been forced to marry. In terms of the number of children, the sample group was as follows: without children (35.67%), with one child (33.33%), with two children (23.57%), and with three children and more (7.43%).

## Haj-Yahia’s questionnaire of violence against women

The Haj-Yahia’s questionnaire of Violence against Women consisted of 32 items and 4 factors: the first factor, which included items 1–16, measures psychological abuse; the second factor, which includes items 17–27, assesses physical abuse; the third factor, which includes items 28–30, evaluates sexual abuse, and the fourth factor, which includes items 31 and 32, measures economic abuse. The questionnaire is designed as a dichotomous scale (0 = never and 1 = at least once). Then cumulative scores were calculated for each pattern of abuse. Psychological abuse was assessed as follows: “never” (the wife had never been subjected to any of these acts), “mild” (the wife had been subjected to 1–5 acts), “moderate” (the wife had been subjected to 6–10 acts), and “severe” (the wife had been subjected to 11 or more acts). Physical violence was assessed according to two levels: “never” (the wife had never been subjected to any of these acts) and “at least once” (the wife had been subjected to at least one of these acts). Sexual abuse and economic abuse were measured according to the same approach (i.e., “never” and “at least once”). Cronbach’s alpha coefficients for the four factors of the Haj-Yahia’s questionnaire were 0.92, 0.93, 0.86, and 0.71, respectively [15].

## Cross-cultural adaption to Persian

To evaluate the translation validity of the questionnaire, the Backward-Forward method was used as a guide for cross-cultural adaptation of health-related questionnaires [20]. The English version of Haj-Yahia’s questionnaire of Violence against Women was prepared and then translated by two subject experts related to the subject and two Persian versions were obtained independent of the main questionnaire. The difference between the Persian versions was examined and a final Persian version was presented. In the next step, the Persian version of the questionnaire was translated into English by two bilingual experts fluent in English, and the second version of the questionnaire was prepared. The compatibility of the second version with the original one was carefully examined and after receiving the suggestions, the necessary modifications were made in the Persian version and finally the third version was presented. The Persian version of the questionnaire can be found in Additional file 1.

## Statistical analyses

A quantitative method was used to evaluate face validity. To determine the face validity, the impact score of each question was calculated. To assess the impact scores, the questionnaire was completed by 20 married women, to determine the importance of each of the 32 questions based on a five-point Likert scale (absolutely important (score 5), important (score 4), moderately important (score 3), slightly important (score 2) and not important at all (score 1)). The impact score was calculated according to the following formula:

$$\mathrm{Impact}\;\mathrm{score}\:=\:\mathrm{Frequency}\;(\%)\;\times\;\mathrm{Importance}.$$

Frequency is the ratio of people who gave the questions a score of 4 and 5, and the importance is the average score of the respondents based on the desired Likert scale. Only questions with a score equal to or greater than 1.5 are acceptable [21].

Content validity was also quantitatively evaluated using content validity ratio (CVR) and content validity index (CVI). In determining the content validity ratio, a group of experts, which consisted of 8 psychiatrists, evaluated each item with three options (necessary, useful but unnecessary, and unnecessary). Responses were calculated based on the CVR formula, adapted to the Lawshe Table [22] and finally, numbers equal to and above 0.75 were accepted. After determining and calculating the CVR, the CVI was measured based on Waltz and Basel’s method [23]. To do so, the questionnaire was given again to the eight psychiatrists to calculate CVI and they were asked to comment on the relevancy, clarity, and simplicity of each of the 32 questions based on a four-part Likert scale (1: unrelated, 2: slightly related, 3: related, and 4: completely related). For this purpose, CVI was computed as the number of experts giving the rating 3 and 4 to each item, divided by the total number of experts [24]. Hyrkas et al. (2003) recommended a score of 0.79 and above for accepting items based on a CVI score [25]. In the next step, based on the average of CVI scores of all the items, the average content validity index (S-CVI / Ave) was calculated. Polit and Beck (2006) recommended a score of 0.90 or higher as acceptable S-CVI/ Ave [24].

Interclass correlations (ICC) were calculated in a sample of 40 married women after 21 days to examine the temporal stability. If ICC is higher than 0.80, the rate of stability is desirable [26]. Cronbach’s alpha coefficient and McDonalds’ Omega were used to examine internal consistency.

In confirmatory factor analysis, according to the Rule of 10, ten respondents were required for each latent variable [27]. Considering the number of factors (latent variables) of the questionnaire in the present study, the sample size was sufficient to perform factor analysis. Since all variables were categorical, the weighted least square mean and variance adjusted (WLSMV) was used. Several model fit indices and their criteria were used to examine the goodness-of-fit of the Haj-Yahia’s four-factor model: Tucker-Lewis Index (TLI), comparative fit index (CFI), root mean square error of approximation (RMSEA), and Standardized Root Mean Squared Residual (SRMR).

We performed confirmatory factor analysis using Mplus version 8 software and for other calculations, we used STATA V14.

## Results

### Descriptive statistics and tetracuric correlations

Descriptive statistics and correlation coefficients between the variables observed in Table 1 are presented. Item 2 (Yelled at you during a heated argument?) demonstrated the largest mean (M = 0.71, SD = 0.46) among the variables. In contrast, Item 27 (Attacked you with a dangerous implement such as a knife or metal rod?) was associated with the smallest mean and the smallest standard deviation scores (M = 0.07, SD = 0.26). Although there was a statistically significant and positive relationship between all items of the questionnaire (*p* < .05), the highest correlation coefficients were among the items of physical violence (*r* = .76 to 0.95).

**Table 1:** Descriptive Statistics and Tetrachoric Correlation Coefficients among Observed Variables

Items	Mean	SD	1	2	3	4	5	6	7	8	9	10	11	12	13	14	15	16	17	18	19	20	21	22	23	24	25	26	27	28	29	30	31	32
**Q1**	0.53	0.50	-																															
**Q2**	0.71	0.46	0.48	-																														
**Q3**	0.44	0.50	0.48	0.75	-																													
**Q4**	0.46	0.50	0.39	0.54	0.62	-																												
**Q5**	0.41	0.49	0.45	0.70	0.66	0.64	-																											
**Q6**	0.38	0.49	0.26	0.71	0.58	0.37	0.54	-																										
**Q7**	0.26	0.44	0.51	0.45	0.52	0.65	0.63	0.39	-																									
**Q8**	0.35	0.48	0.41	0.77	0.78	0.62	0.74	0.63	0.59	-																								
**Q9**	0.48	0.50	0.36	0.52	0.58	0.57	0.60	0.39	0.71	0.62	-																							
**Q10**	0.40	0.49	0.35	0.65	0.74	0.53	0.54	0.54	0.56	0.70	0.64	-																						
**Q11**	0.55	0.50	0.25	0.38	0.48	0.36	0.59	0.34	0.42	0.57	0.51	0.52	-																					
**Q12**	0.24	0.43	0.34	0.65	0.70	0.54	0.68	0.61	0.54	0.77	0.63	0.72	0.50	-																				
**Q13**	0.27	0.45	0.43	0.52	0.52	0.52	0.57	0.45	0.45	0.56	0.53	0.55	0.57	0.60	-																			
**Q14**	0.37	0.48	0.43	0.70	0.70	0.54	0.62	0.55	0.64	0.65	0.69	0.80	0.67	0.75	0.49	-																		
**Q15**	0.43	0.50	0.43	0.62	0.69	0.55	0.64	0.50	0.54	0.71	0.61	0.78	0.57	0.80	0.52	0.82	-																	
**Q16**	0.34	0.47	0.44	0.50	0.62	0.62	0.55	0.40	0.60	0.59	0.61	0.55	0.44	0.68	0.43	0.65	0.73	-																
**Q17**	0.29	0.45	0.32	0.69	0.56	0.55	0.69	0.58	0.52	0.60	0.53	0.53	0.33	0.64	0.55	0.57	0.57	0.52	-															
**Q18**	0.29	0.45	0.38	0.61	0.58	0.58	0.68	0.55	0.62	0.61	0.54	0.70	0.49	0.62	0.61	0.63	0.62	0.62	0.86	-														
**Q19**	0.29	0.45	0.32	0.67	0.56	0.55	0.61	0.48	0.60	0.58	0.56	0.62	0.50	0.64	0.52	0.62	0.57	0.50	0.84	0.95	-													
**Q20**	0.09	0.29	0.35	0.69	0.65	0.43	0.69	0.53	0.58	0.65	0.52	0.64	0.56	0.68	0.59	0.60	0.64	0.61	0.83	0.89	0.83	-												
**Q21**	0.33	0.47	0.32	0.61	0.65	0.56	0.65	0.49	0.53	0.51	0.46	0.58	0.40	0.66	0.46	0.52	0.50	0.50	0.81	0.88	0.83	0.80	-											
**Q22**	0.25	0.43	0.24	0.59	0.65	0.45	0.63	0.47	0.58	0.54	0.50	0.57	0.46	0.65	0.57	0.59	0.48	0.49	0.84	0.91	0.88	0.88	0.87	-										
**Q23**	0.13	0.34	0.30	0.64	0.69	0.53	0.68	0.51	0.58	0.57	0.53	0.74	0.53	0.84	0.70	0.64	0.61	0.59	0.86	0.88	0.84	0.93	0.89	0.92	-									
**Q24**	0.14	0.35	0.25	0.63	0.66	0.61	0.61	0.51	0.51	0.55	0.52	0.70	0.48	0.70	0.52	0.63	0.55	0.54	0.80	0.85	0.90	0.80	0.81	0.90	0.86	-								
**Q25**	0.21	0.41	0.24	0.68	0.65	0.57	0.65	0.33	0.57	0.64	0.47	0.58	0.42	0.66	0.48	0.57	0.44	0.49	0.79	0.89	0.86	0.87	0.93	0.90	0.89	0.92	-							
**Q26**	0.10	0.30	0.41	0.64	0.67	0.53	0.69	0.50	0.63	0.53	0.51	0.62	0.35	0.67	0.64	0.62	0.66	0.52	0.90	0.82	0.82	0.88	0.78	0.82	0.93	0.77	0.87	-						
**Q27**	0.07	0.26	0.20	0.55	0.67	0.53	0.62	0.54	0.51	0.57	0.47	0.68	0.61	0.62	0.66	0.75	0.61	0.62	0.78	0.87	0.82	0.97	0.79	0.86	0.86	0.94	0.76	0.91	-					
**Q28**	0.32	0.47	0.24	0.44	0.54	0.37	0.41	0.37	0.37	0.42	0.45	0.49	0.48	0.64	0.39	0.47	0.38	0.36	0.46	0.50	0.50	0.63	0.49	0.56	0.65	0.60	0.70	0.60	0.68	-				
**Q29**	0.19	0.39	0.33	0.43	0.55	0.51	0.60	0.42	0.36	0.49	0.56	0.51	0.39	0.64	0.46	0.57	0.59	0.53	0.51	0.47	0.55	0.59	0.38	0.42	0.56	0.60	0.50	0.53	0.60	0.68	-			
**Q30**	0.34	0.47	0.29	0.47	0.55	0.49	0.46	0.39	0.46	0.45	0.52	0.57	0.53	0.64	0.46	0.49	0.44	0.42	0.42	0.48	0.46	0.70	0.45	0.47	0.69	0.60	0.60	0.63	0.78	0.94	0.65	-		
**Q31**	0.24	0.43	0.38	0.53	0.62	0.60	0.67	0.52	0.56	0.50	0.59	0.61	0.45	0.69	0.45	0.66	0.63	0.58	0.47	0.50	0.56	0.73	0.50	0.51	0.70	0.64	0.57	0.71	0.76	0.66	0.61	0.62	-	
**Q32**	0.22	0.42	0.41	0.54	0.68	0.57	0.61	0.49	0.61	0.58	0.56	0.58	0.46	0.62	0.55	0.63	0.52	0.63	0.46	0.64	0.64	0.73	0.53	0.62	0.74	0.67	0.61	0.71	0.75	0.54	0.57	0.56	0.86	-

## Face validity and content validity

Impact score results indicated that all questions had a score equal to or higher than 1.5, so they were retained in the questionnaire. Therefore, it can be concluded that the questionnaire was simple and understandable. The CVI results indicated that all questions had a score higher than 0.79 and therefore, were considered appropriate. It is worth mentioning that the average content validity index (S-CVI /Ave) was 0.96. Polit and Beck (2006) recommended a score of 0.90 or higher as acceptable S-CVI /Ave (Scale-Level CVI /Average). The CVR results demonstrated that all questions were equal to or higher than the Lawshe Table number (0.75), implying that all the necessary and important questions were included in this questionnaire.

## Confirmatory factor analysis

**Fig. 1:**
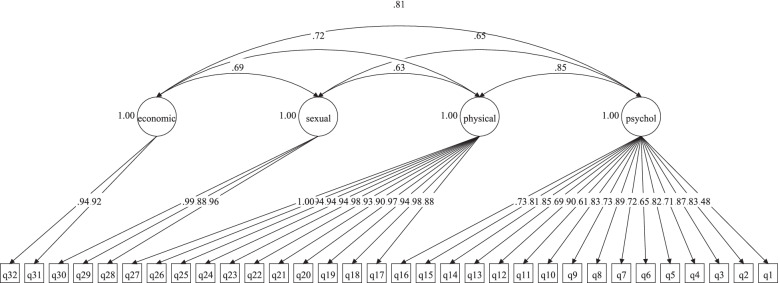
Confirmatory factor analysis of the Haj-Yahia’s four-factor model

Confirmatory Factor Analysis for Haj-Yahia’s four-factor model is presented in Fig. 1. All items had statistically significant loadings onto their latent factor (< 0.001). According to the results of Table 2, the model fit indices were as follows: TLI = 0.986, CFI = 0.987, RMSEA = 0.039 and SRMR = 0.057.

**Table 2:** Goodness of fit indices for Haj-Yahia’s four-factor model

Model	χ2 (df)	χ2/df	TLI	CFI	SRMR	RMSEA (90%CI)
Model (4 Factor)	**780.416 (458)**	**1.70**	**0.986**	**0.987**	**0.057**	**0.039 (0.034–0.043)**

*χ2 (df)*: Chi-square statistics (degree of freedom), *CFI* comparative fit index, *TLI* Tucker-Lewis Index; *SRMR* standard root mean square residual, *RMSEA* root mean square error of approximation, *CI* confidence interval

## Reliability

To determine the internal consistency, Cronbach’s alpha coefficient and McDonalds’ Omega were calculated in a sample of 471 married women, which was 0.95 for the total questionnaire according to Table 3. Using Cronbach’s alpha coefficient, the internal consistency of psychological abuse, physical abuse, sexual abuse, and economic abuse was found to be 0.90, 0.93, 0.79, and 0.78 respectively. In addition, using McDonalds’ Omega, internal consistency estimates of 0.90, 0.94, 0.84, and 0.76 were found for the four factors respectively. The ICC rate for the total questionnaire was 0.98 and for different dimensions of the questionnaire ranged from 0.93 to 0.99, which was in the acceptable range.

**Table 3:** Cronbach’s alpha coefficient and intra-cluster correlation, confidence interval, and significant probability

Component	Cronbach’s alpha	McDonalds’ Omega	ICC	CI= %95	*P*-value
				**Lower Bound Upper Bound**	
Psychological	0.90	0.90	0.97	0.72 0.99	0.0001
Physical	0.93	0.94	0.99	0.96 0.99	0.0001
Sexual	0.79	0.84	0.93	0.83 0.97	0.0001
Economic	0.78	0.76	0.94	0.89 0.97	0.0001
Wife abuse (total)	0.95	0.95	0.98	0.69 0.99	0.0001

## Discussion

Domestic violence is a chronic life-threatening disease that, if left untreated, increases in severity and frequency and leads to serious adverse effects to health in women. Studies have shown that identifying victims of violence through screening and providing counseling and support services to them can improve the quality of life and reduce violence-related injuries in married women who suffer from violence [28, 29]. Therefore, this study aimed to examine the psychometric properties and factor structure of the Persian version of the Haj-Yahia’s questionnaire in a large sample of married Iranian women. The findings suggest that the Persian version of the Haj-Yahia questionnaire showed good psychometric properties. This tool will help assess violence among married women.

The main methods used for determining the content validity in the studies of instruments’ psychometric properties have been CVI and CVR which had desirable values in the present study and were consistent with the previous studies [30, 31].

In this study, we examined whether a new dataset of married women is appropriate for the 4-factor model devised in a previous study [15]. For that, CFA was performed and model fits were examined. To examine the model fit of CFA, we have to consider the different fit indices of the model. It has been suggested that for the RMSEA index, values less than 0.05 have a good fit, and values between 0.05 and 0.08 have an acceptable fit [32]. Therefore, a value of 0.039 in our sample shows a good fit. Also, the CFI and TLI values were 0.99, which indicates a good fit [33]. The values of CFI ≥ 0.95, TLI ≥ 0.95, and RMSEA ≤ 0.10 were recommended by Meyers et al.[34]. As a result, based on the values of the desired indices, this sample has a good and acceptable fit with Haj-Yahia’s four-factor model.

Haj-Yahia’s questionnaire included four factors of psychological violence, physical violence, sexual violence, and economic violence. Psychological violence has manifested itself in insults, threats, humiliation, and verbal abuse. Physical violence is when a person hurts or tries to hurt a partner by slapping, pushing, squeezing a person’s throat, hitting, or using another type of physical force, and sexual violence is any sexual act or attempts to obtain a sexual act by violence or coercion [35, 36]. Economic violence is any act or behavior which causes economic harm to an individual. Economic violence can take the form of, for example, property damage, restricting access to financial resources, or not complying with economic responsibilities [37]. Economic violence is an important aspect of violence against women that has been ignored in many studies. for example, the oldest Iranian scale in this field focused on assessing physical, mental, and sexual abuse using 44 items [17]. In addition, the Violence Against Women Instrument (VAWI), often used to assess domestic violence, assesses three dimensions of violence, including psychological, physical, and sexual [38].

The results indicated a strong Cronbach’s alpha value for the four subscales (psychological, physical, sexual, and economic), which shows that these four subscales and the total scale are reliable enough for a Tehran or Iran sample. The results are consistent with the results of Indo et al. [39]. The intra cluster correlation coefficient in this study was 0.98 for all the items and was in the range of 0.93 to 0.99 for the dimensions of the questionnaire. This result shows that the reliability coefficient of this tool is excellent in this method as Croon has introduced the inter-cluster correlation coefficient of 0.75-1 as the excellent level [40].

The results obtained from this study were similar to those reported in the Palestinian study [15], which showed that Haj-Yahia’s questionnaire has good cross-cultural construct validity and good internal reliability. However, more studies are needed in different populations and cultures to examine these and other psychometric properties.

We can point out the diversity of the dimensions of the present questionnaire as well as its short form compared with other tools such as the Conflict Resolution Tactics Questionnaire (Panaghi et al.), and Spouse Abuse Questionnaire (Ghahari et al.,2006). In addition, Panaghi et al.‘s questionnaire is mainly suggested for clinical settings and the Conflict Resolution Tactics questionnaire has not specifically addressed the types of wife abuse.

This study had several limitations. The research sample has been selected by convenience sampling, so its generalization to the whole community should be done carefully. The content of the questionnaire’s items also evaluates the personal and confidential information related to the respondents. Therefore, subjects may be cautious in completing the questionnaire, which causes damages to the validity of the questionnaire.

## Conclusions

Lack of appropriate tools to measure wife abuse is one of the most important problems of researchers working in this field. Appropriate and specific tools can play a significant role in the field of preventing abuse, consulting, and facilitating psychosocial interventions. Given the importance of this issue, one of the first requirements for interventions in the field of wife abuse is access to tools that have good validity and reliability and its questions have a cultural fit with the target group and society. According to the results of this study, the Persian version of Haj-Yahia’s Questionnaire has these criteria and evaluates various aspects of wife abuse and it is suggested to be used as a suitable tool in survey studies or to evaluate the impact of wife abuse interventions.

## Supplementary Information

**Additional file 1.**

## Data Availability

An aggregated version of the data might be made available by the corresponding author on reasonable request with permission from the ethics committee.

## Abbreviations

CVI: Content Validity Index
CVR: Content Validity Ratio
I-CVI: Item-Level CVI
S-CVI/Ave: Scale-Level CVI /Average
IPF: Iterated Principal Factor Analysis
