# Supplementary material for: Psychometric properties of Haj-Yahia’s questionnaire of violence against women in a sample of married women in Tehran, Iran
Source: BMC Public Health. 2022 Mar 7;22:448. doi: 10.1186/s12889-022-12831-8 (PMC8900410; doi:10.1186/s12889-022-12831-8)
Supplement: Supplementary file 1 — Additional file 1. [file 12889_2022_12831_MOESM1_ESM.docx]

**Table A1.** Persian version of the items of Haj-Yahia’s questionnaire of violence against women

| **حداقل یکبار** | **هرگز** | **درطول 12 ماه گذشته همسر شما...** |
| --- | --- | --- |
|  |  | 1- مشاجره را خاتمه داده و درباره موضوعی که به هر دوی شما مربوط است شخصا تصمیم گرفته است؟ |
|  |  | 2- حین یک مشاجره تند بر سر شما فریاد کشیده است؟ |
|  |  | 3- شما را با اسامی و القاب ناپسند، توهین، ناسزا، و فحاشی خطاب قرار داده است؟ |
|  |  | 4- سعی نموده تا شما را از انجام کار مورد علاقه خودتان باز دارد؟ |
|  |  | 5- طوری به شما نگاه کرد که شما را وحشت زده کند ؟ |
|  |  | 6- پس از یک مشاجره با حالت خشمگین و در حالی که به شما ناسزا گفته و بر سر شما فریاد کشیده منزل را ترک کرده است؟ |
|  |  | 7- سعی کرده از طریق پرس و جو، بازجویی و تعقیب رفتار شما را کنترل نماید؟ |
|  |  | 8- تهدید کرد که چیزی به طرف شما پرتاب می کند و یا با کلام شما را مورد تهدید قرار داده است؟ |
|  |  | 9- شما را متهم کرده که بیش از آنچه که به او اهمیت می دهید به دیگران بها داده و به آنها اهمیت می دهید؟ |
|  |  | 10- به منظور تهدید، شما و یا اطرافیان شما را مورد تحقیر و یا توهین قرار داده است؟ |
|  |  | 11- شما را متهم به تنبلی، بی تفاوتی، و کوتاهی در انجام امور منزل و امور مربوط به خودش کرده است؟ |
|  |  | 12- انجام کاری را از شما درخواست نموده و یا شما را مجبور به انجام کاری کرده تا بدین وسیله شما را مورد توهین و تحقیر قرار دهد؟ |
|  |  | 13- شیوه ی تربیتی شما در قبال فرزندان را دست کم گرفت و شما را متهم به کوتاهی در انجام وظایف به عنوان مادر و همسر کرده است؟ |
|  |  | 14- با انتقاد و سرزنش خانواده، خویشان و یا دوستان شما را تحقیر نموده است؟ |
|  |  | 15- با تحقیر افکار، عقاید، نگرش و رفتارتان، شما را مورد سرزنش و عیب جویی قرار داده است؟ |
|  |  | 16- طرز پوشش، اندام و ظاهر شما را تحقیر کرده است؟ |
|  |  | 17- در زمان مشاجره با شما چیزی را پرتاب نموده، لگد زده و یا شکسته است؟ |
|  |  | 18- شما را هل داده، لگد زده و یا تلاش نموده تا شما را با مشت مورد ضرب و جرح قرار دهد؟ |
|  |  | 19- شما را به شدت هل داده یا کشیده است؟ |
|  |  | 20- شما را با چاقو و یا هر وسیله تیز دیگر تهدید نموده است؟ |
|  |  | 21- به شما سیلی زده است؟ |
|  |  | 22- با دست ضربات محکمی را بر قسمت های مختلف از بدنتان وارد نموده است؟ |
|  |  | 23- با چوب، کمربند، و یا وسیله مشابه دیگری به شما حمله کرده است؟ |
|  |  | 24- سعی نموده شما را خفه کند و یا با حلقه کردن بازوهایش به دورگردنتان به شما آسیب برساند؟ |
|  |  | 25- موها و یا لباسهای شما را کشیده است؟ |
|  |  | 26- با وسایل و لوازم خانه (برای مثال صندلی) به شما حمله کرده است؟ |
|  |  | 27- با وسیله ای خطرناک مانند چاقو و یا میله فلزی به شما حمله کرده است؟ |
|  |  | 28- سعی نموده تا بدون رضایت شما رابطه ی جنسی با شما برقرار نماید؟ |
|  |  | 29- عدم رضایت خود از رابطه ی جنسی با شما را به صورت تحقیر آمیز و سرزنش کننده ای ابراز کرده است؟ |
|  |  | 30- بدون رضایت شما رابطه ی جنسی با شما داشته است؟ |
|  |  | 31- شما را از استفاده از پول خانواده به آن صورت که صلاح می دانید منع کرده است؟ |
|  |  | 32- با سوء استفاده از پول و منابع مالی خانواده سعی کرده رفتار شما را کنترل نموده و یا شما را مجبور نموده تا آنچه را که او می خواهد، انجام دهید؟ |

**Table A2.** English version of the items of Haj-Yahia’s questionnaire of violence against women

| **During the last 12 months your husband...** | **Never** | **At least once** |
| --- | --- | --- |
| 1- Finished an argument and made his own decision about a matter that concerns both of you? |  |  |
| 2- Yelled at you during a heated argument? |  |  |
| 3- Insulted you, cursed you, used abusive language, or called you names? |  |  |
| 4. Tried to prevent you from doing what you want (e.g., visiting relatives or friends)? |  |  |
| 5- Given you dirty looks in an attempt to intimidate you? |  |  |
| 6- Stormed out of the house after an argument, cursing and yelling at you? |  |  |
| 7- Tried to control your behavior by investigating, interrogating, and following you? |  |  |
| 8- Threatened to throw something at you and said things to intimidate you? |  |  |
| 9- Accused you of paying more attention to and doing more for others than for him? |  |  |
| 10- Degraded and insulted you or your acquaintances in an attempt to intimidate you? |  |  |
| 11- Accused you of being lazy, indifferent, and failing to fulfill your obligations toward him and the household? |  |  |
| 12- Requested or forced you to do something with the intention of insulting or humiliating you? |  |  |
| 13- Belittled your approach toward child rearing or accused you of being a failure as a wife and mother? |  |  |
| 14- Degraded your family, relatives, or friends by humiliating or cursing them? |  |  |
| 15- Reprimanded or scolded you while belittling your thoughts, beliefs, and attitudes**?** |  |  |
| 16- Belittled the way you dress, your body, and the way you keep up your appearance? |  |  |
| 17- Thrown, kicked, or broken something while arguing with you? |  |  |
| 18- Pushed you, kicked you, or tried to knock you over? |  |  |
| 19- Pushed or pulled you hard? |  |  |
| 20- Threatened you with a knife or another sharp implement? |  |  |
| 21- Slapped you? |  |  |
| 22- Attacked you with his hands on different parts of your body? |  |  |
| 23- Attacked you with a stick, a belt, or another object of that kind? |  |  |
| 24- Tried to choke you or placed his arms around your neck in an attempt to harm you? |  |  |
| 25- Pulled your hair or yanked your clothes? |  |  |
| 26- Attacked you with household equipment (e.g., a chair)? |  |  |
| 27- Attacked you with a dangerous implement such as a knife or metal rod? |  |  |
| 28- Tried to have sex with you without your consent? |  |  |
| 29- Expressed dissatisfaction with your sexual relationship in an insulting and degrading way? |  |  |
| 30- Had sex with you without your consent? |  |  |
| 31- Prevented you from using the family's money as you see fit? |  |  |
| 32- Tried to control your behavior or force you to do what he wants, while misusing the family's income and other resources to do so? |  |  |
